# Medical School to Residency: How Can We Trust the Process?

**DOI:** 10.7759/cureus.14485

**Published:** 2021-04-14

**Authors:** Gary L Beck Dallaghan, Irene Alexandraki, Jennifer Christner, Meg Keeley, Sorabh Khandelwal, Beat Steiner, Paul A Hemmer

**Affiliations:** 1 Office of Medical Education, University of North Carolina at Chapel Hill School of Medicine, Chapel Hill, USA; 2 Medicine, Florida State University College of Medicine, Tallahassee, USA; 3 Pediatrics, Baylor University College of Medicine, Houston, USA; 4 Pediatrics, University of Virginia School of Medicine, Charlottesville, USA; 5 Emergency Medicine, The Ohio State University College of Medicine, Columbus, USA; 6 Family Medicine, University of North Carolina School of Medicine, Chapel Hill, USA; 7 Medicine, Uniformed Services University of the Health Sciences, Bethesda, USA

**Keywords:** medical student performance evaluation, mspe, medical students, residency training

## Abstract

Background

To say that the transition from undergraduate medical education (UME) to graduate medical education (GME) is under scrutiny would be an understatement. Findings from a panel discussion at the 2018 Association of American Medical Colleges Annual meeting entitled, “Pass-Fail in Medical School and the Residency Application Process and Graduate Medical Education Transition” addressed what and when information should be shared with residency programs, and how and when that information should be shared.

Materials and Methods

Over 250 participants representing UME and GME (e.g. leadership, faculty, medical students) completed worksheets addressing these questions. During report-back times, verbal comments were transcribed in real time, and written comments on worksheets were later transcribed. All comments were anonymous. Thematic analysis was conducted manually by the research team to analyze the worksheet responses and report back comments.

Results

Themes based on suggestions of what information should be shared included the following: 1) developmental/assessment benchmarks such as demonstrating the ability/competencies to do clinical work; 2) performance on examinations; 3) grades and class ranking; 4) 360 evaluations; 5) narrative evaluations; 6) failures/remediation/gaps in training; 7) professionalism lapses; 8) characteristics of students such as resiliency/reliability; and 9) service/leadership/participation. In terms of how this information should be shared, the participants suggested enhancements to the current process of transmitting documents rather than alternative methods (e.g., video, telephonic, face-to-face discussions) and information sharing at both the time of the match and again near/at graduation to include information about post-match rotations.

Discussion

Considerations to address concerns with the transition from medical school to residency include further enhancements to the Medical Student Performance Evaluation, viewing departmental letters as ones of evaluation and not recommendation, a more meaningful educational handoff, and limits on the number of residency applications allowed for each student. The current medical education environment is ready for meaningful change in the UME to GME transition.

## Introduction

The transition from undergraduate medical education (UME) to graduate medical education (GME) has been an issue for several years [1]. In the past, the relationship between UME and GME was more like a courtship, where medical students and residency program directors (PD) sought the right match to a program. With the ever-increasing numbers of applicants, PDs face challenges in narrowing the pool of potential candidates to invite for interviews. As a result of the 2020 pandemic, traditional approaches to meet and screen students have also been hampered [2,3].

The Electronic Residency Matching Program (ERAS) process encourages students to apply to a vast number of programs, often overwhelming an individual program’s ability to effectively screen for and identify qualified applicants. This, in turn, leads to an emphasis on licensing examination scores as a proxy for a “good student”. This reality is driving anxiety and behaviors to maximize test scores. Students are relentlessly pursuing honors in clinical rotations, scheduling months of “audition rotations” in their fourth year, and spending large amounts of money traveling across the country for numerous interviews [4].

The recent Invitational Conference on USMLE Scoring (InCUS) brought residency matching issues into sharper focus and provided the medical education community with a thorough summary of the issues [5]. The bold decision to make USMLE (United States Medical Licensing Examination) Step 1 a Pass/Fail examination was welcomed by some and criticized by others. The variability in grading frameworks across medical schools as well as within schools causes concerns for PDs [6]. One study identified as many as 19 different grading schemes used by medical schools, where top-ranked medical schools awarded a higher proportion of the highest grades to ensure students were perceived favorably [7].

Attempting to use narrative comments associated with clinical clerkship is also challenging. Language used to describe performance varies across schools as well as within schools [1,8]. Additionally, a recent study identified racial and ethnic disparities not only in standardized testing but also in narrative comments [9]. Multiple grading schemes and lack of uniform performance descriptions result in a lack of trust by PDs in the accuracy of the Medical Student Performance Evaluation (MSPE).

The MSPE has had varying importance as part of the residency application and selection process [10]. Only 70% of medical schools used school-wide comparative performance data in their MSPEs in 2017 [11]. In the 2018 survey of Program Directors in Internal Medicine, respondents asked for more transparency and comparative data and for information on areas where a candidate needs to improve [12]. The MSPE often only covers the core clerkship year, which is happening earlier with UME curricular reform [13], and perhaps a few advanced clinical rotations, and therefore PDs do not receive data on performance and achievement in the final months of medical school.

Medical schools have strong incentives to ensure that their students obtain residency placements so that they may have motivation to hide or minimize student issues. Percentage match rates affect school administrators’ performance reviews and the reputation of the medical school. The MSPE is difficult to interpret as grading structures vary and often use coded language that is inconsistent from school to school [1,8]. In addition, obscure or vague narrative comments and omission of academic difficulties have contributed to the tension between UME and GME and lack of trust in the process [14].

Based on this discussion, it is no surprise that there is an intensity of interest in the UME to GME transition unlike what many of us have seen in our careers [15]. All stakeholders including medical students continue to face uncertainties going forward. The Alliance for Clinical Education (ACE) conducted a workshop to further explore this issue at the Association of American Medical Colleges (AAMC) Learn Serve Lead meeting in 2018. Our aim was to examine participants’ perspectives on how the MSPE can be better used to complement the Match process in light of various grading schemas by many medical schools.

## Materials and methods

ACE presented a panel discussion entitled “Pass-Fail in Medical School and the Residency Application Process and Graduate Medical Education Transition” at the 2018 AAMC Learn Serve Lead meeting with over 250 medical educators. ACE is an organization that is comprised of representatives from eight medical education organizations (emergency medicine, family medicine, internal medicine, neurology, obstetrics and gynecology, pediatrics, psychiatry, and surgery). The objectives of the session were to:

1) recognize current trends in UME grading, and the challenges this may present in the residency application process,

2) describe approaches UME could take to enhance the quality of information gathered and reported to GME, and

3) describe strategies to enhance the residency review/selection process for medical students who come from programs that do not use traditional grading schemas, such as pass/fail, or honors/high pass/pass/fail.

To address the UME-to-GME transition, we used small group breakouts to answer two questions on a worksheet: 1) what and when should information be shared with residency programs? and 2) how and when should that information be shared? Participants representing UME and GME (e.g. leadership, faculty, medical students) completed worksheets to address these questions. During report-back times, comments were transcribed in real time into the slide presentation. Any comments participants made during the debrief session were also transcribed and kept anonymous. The worksheets did not include any personal or other identifiable information.

Data analysis

We used thematic analysis to identify patterns within and across the collected data to construct meaning [16]. We combined comments from the completed worksheets and the debrief session into a single document. We worked in pairs following an iterative approach to identify emerging themes. Two conference calls were held to discuss our interpretation of themes and their interconnectedness and resolve any differences, and we reached consensus on the final themes. This study was approved by the Institutional Review Board at the Uniformed Services University of the Health Sciences as exempt.

## Results

The space reserved for this session was designed to accommodate 250 participants. Additional accommodations were needed for people standing in the back of the room; however, a formal count was not taken due to the size and layout of the room. Participants included assistant or associate deans, department chairs, clerkship directors, designated institutional officials, and PDs. They consisted primarily of professors or associate professors. A total of 61 worksheets were returned with three to four participants working together. Results of the thematic analysis are presented and addressed in the context of existing literature. We summarized themes in Table 1.

**Table 1:** UME-to-GME Themes: What to Share in a Competency-based Medical Education Model GME, graduate medical education; UME, undergraduate medical education

What is the Information to Share from UME to GME	HOW to Share the Information from UME to GME
Developmental/assessment benchmarks, such as demonstrating the ability/competencies to do clinical work; exam performance, including standardized exams; grades and class ranking 360 evaluations; narrative evaluations; failures/remediation/gaps in training; professionalism lapses; personal characteristics of students, such as resiliency/reliability; Service/leadership	Further standardization of the Medical Student Performance Evaluation (MSPE); standardized letters of evaluation; educational hand-offs; limits on electronic applications

UME-to-GME information to share

Themes based on suggestions of what to share during the initial application process included the following: 1) developmental/assessment benchmarks, such as demonstrating the ability/competencies to do clinical work; 2) exam performance, including standardized exams; 3) grades and class ranking; 4) 360 evaluations; 5) narrative evaluations; 6) failures/remediation/gaps in training; 7) professionalism lapses; 8) personal characteristics of students, such as resiliency/reliability; and 9) service/leadership.

Consequently, the themes about what information should be shared after the residency application were largely the same. Participants did indicate a greater emphasis on reporting failures and/or remediation requirements for clerkships be communicated clearly.

How to share the information resulted in themes focused on transparency and conciseness of information. Participants wrote there needs to be “honesty in the MSPE” and “…gaps in someone’s training (red flag)…” The participants felt that the current MSPE is a sufficient summary of student performance but is lacking in its honest appraisal of students’ strengths and weaknesses. One participant commented, “We don’t need a different mechanisms or method for sharing, just improve the current tool.”

Improving the UME-to-GME transition

Four themes pertained to improving the transition, which included further standardization of the MSPE, standardized letters of evaluation, educational hand-offs, and limits on electronic applications.

Further Standardization of the MSPE

Considering the variability that exists in the curricular and assessment structures among medical schools and the move to pass/fail for USMLE Step 1, it will be important to continue efforts to standardize the reporting of student performance and academic progress in the MSPE across medical schools. Specific recommendations pulled from worksheets included: “narrative from clerkships, clerkship key metrics (shelf, clinical performance), narrative from mentors” and “…more quotes from each clerkship director.”

Conversely, participants suggested that narrative comments may not be sufficient without accompanying metrics. “I don’t trust the narrative unless supplemented by how they compare to peers in their class (to their letters of support in packet compared to peers or some discriminating factor).”

Standardized Letters of Evaluation

Participants discussed the use of more comprehensive letters of evaluation such as those developed by emergency medicine. Emergency Medicine (EM), in a process that started in 1995, has developed a standardized letter of evaluation (SLOE) to improve trustful communication between UME and GME and is intended to augment the MSPE and provide more useful information than the traditional letter of recommendation [17]. Additionally, it was suggested that programs be transparent with how they prioritize letters of support, e.g. “clerkship attending, department chair, MSPE.”

Educational Hand-off

Another proposed enhancement is an educational handover of information from UME to GME programs to allow for a competency-based continuum of learning. In the standard residency match schedule, the MSPE is completed in September to be released on October 1, leaving several months of experience undocumented. A formalized educational handover could be designed as a final individualized learning plan for residency [18]. Participants suggested this could reflect discipline-specific milestone performance as well as core entrustable professional activities for entering residency based on additional student performance evaluations and end of medical school assessments. Various modes of delivery were also suggested, such as a handoff letter to the matched program or having the graduating student deliver the letter at orientation.

Limits on Electronic Applications

Residency program selections are overwhelmed with application numbers from students who are over-applying based on some unfounded fears regarding the Match landscape. Participants in the GME space voiced their strong desire to have a more holistic approach to reviewing applications but feel compelled to use examination score cut-offs given the sheer volume of applications. As one participant noted, “does this [application limits] resolve application density without giving students more insight into which programs will be good fits (as one idea to help narrow student focus)?” For this reason, it was suggested to “limit to 20 the number of applications to residency.”

## Discussion

Based on results from the session ACE conducted, participants identified key items that medical schools need to share with residency programs. These items align with recommendations from the MSPE Task Force [19]. It was also felt that the MSPE is a sufficient instrument; however, greater adherence to reporting student gaps and professionalism issues needs to be included. Without transparency, participants noted that they lack trust in the information supplied in the MSPE.

For trust to exist, positive expectations about others facilitate positive behaviors when interacting with them. This results in strengthening positive expectations creating a cycle of expectation and action reinforcing desired outcomes [20]. Trust moderates the relationship between an action and the response [21]. Distrust has been characterized as a lack of confidence in others, unsure of the motives of others [22]. Thus, ranking a medical student based on a glowing MSPE that was found to be misleading leads to distrust. Green et al. noted overt omission of failing or marginal grades in MSPE narratives, which leads to distrust by PDs [23,24].

To enhance trust, clearly articulated details of the grading system each medical school uses, the components of each clerkship grade and their weight, and whether this varies within and/or across phases of the educational program (e.g., from pre-clerkship to clerkship, or within the advanced or fourth-year clerkships) must be reported. With Pass/Fail grading systems being implemented in more medical schools across all four years, there is a need to provide a comparison of each student’s performance in each clerkship with that of his/her class as recommended by the AAMC Task Force [25].

Moreover, any particular student characteristics that stand out in clerkship or course evaluations should be included in the MSPE. Attributes such as professionalism, humanism, and patient-centeredness, which are essential in a well-rounded physician, should be highlighted in the MSPE in a standardized fashion. Although there is now a section for noteworthy characteristics, it is unclear if PDs take these into consideration. In fact, only 37% of respondents to the internal medicine PD survey indicated noteworthy characteristics being influential in rank considerations [12]. Faculty narrative assessments can be valuable in emphasizing student strengths and weaknesses, and there is a greater need for understanding the impact of potential bias in the assessment of learners [14,26].

There should also be transparency and uniformity in the reporting of any lapses in academic progress and professional performance with a description of the issue, any actions taken, and outcomes [27]. These actions may enhance collective trust [20] placed in the MSPE as a reliable summary of student performance. Additionally, receiving feedback from programs on graduates’ performance on core competencies of medical knowledge, patient care, professionalism, communication, practice-based learning, and systems-based practice may also help clarify information included in the MSPE. The AAMC is pilot testing a new process to do this [28].

Although these are good recommendations, there are other systemic issues that impact the transition from UME to GME, the most obvious of which is the inordinate number of applications programs receive. Obstetrics and gynecology residencies have made efforts to control the timing and response process of interview offers, which has elicited a positive response from students and programs, but the priority should be to reduce the overall numbers of applications [29].

The National Resident Matching Program data consistently demonstrate that match rates have not improved with the dramatic increase in applications submitted. The sequential ranks to maximize likelihood of matching vary somewhat by specialty but are well below the inflated number of applications enabled by the ERAS and encouraged by concerns about match rates. The claim that students must submit large numbers of applications in order to secure enough interviews to match only stands based on the fact that other students are over-applying and perpetuating the issue.

Institution of application caps within ERAS perhaps in a phased approach would likely have the most impact on the Match process. This would require programs to share specific and reliable information about their mission and the type of trainee they are looking to match that an applicant could incorporate into their decision with a fixed number of applications. This approach could be coupled with interview offer rounds, fixed dates, and signaling options that would increase match and fill rates. UME and GME would need to prepare for perhaps some decrease in match rates at least initially and alter the Supplemental Offer and Acceptance Program (SOAP) process accordingly as additional rounds of the match process. As a result of the pandemic, ophthalmology programs are putting a cap on the number of virtual interviews they offered in 2020 [30].

Limitations

Limitations of this study stem from data gathered at a large group event at a national meeting. Due to the structure of the AAMC meeting, being able to identify participants and their specific roles in the medical school was not possible. However, given that this meeting draws medical education leaders across the spectrum of training, responses may be considered as expert opinions from various stakeholders.

Suggestions for future study

The results of this study offer suggestions for further study. Little is known about the impact of the MSPE in its current form. More in-depth analysis of different standardized letters of evaluation needs to be undertaken as well. Feed-forward reports after match as well as limiting applications are newer ideas that have yet to be fully investigated and have been recommended by the medical education coalition group as a result of the pandemic. We would challenge medical educators to consider undertaking multi-institutional studies on these recommendations.

## Conclusions

The medical education community finds itself at a crossroads. The announced move to make Step 1 pass-fail, coupled with world events that have created significant challenges to our educational programs, provides a unique opportunity to increase the trust between UME and GME. We can seize this opportunity to forge a vibrant new relationship or we can allow the anxiety from Step 1 simply to shift to Step 2. The findings from our panel discussion presentation provide some insight and guidance to strengthen this valuable relationship so that we can again trust the process and enhance the transition from medical school to residency.
